# Differences in the Optimal Motion of Android Robots for the Ease of Communications Among Individuals With Autism Spectrum Disorders

**DOI:** 10.3389/fpsyt.2022.883371

**Published:** 2022-06-03

**Authors:** Hirokazu Kumazaki, Taro Muramatsu, Yuichiro Yoshikawa, Yoshio Matsumoto, Masaki Kuwata, Keiji Takata, Hiroshi Ishiguro, Masaru Mimura

**Affiliations:** ^1^Department of Future Psychiatric Medicine, Graduate School of Biomedical Sciences, Nagasaki University, Nagasaki, Japan; ^2^National Center of Neurology and Psychiatry, Department of Preventive Intervention for Psychiatric Disorders, National Institute of Mental Health, Tokyo, Japan; ^3^College of Science and Engineering, Kanazawa University, Kanazawa, Japan; ^4^Department of Neuropsychiatry, Keio University School of Medicine, Tokyo, Japan; ^5^Human Augmentation Research Center, National Institute of Advanced Industrial Science and Technology, Chiba, Japan; ^6^Department of Systems Innovation, Graduate School of Engineering Science, Osaka University, Osaka, Japan; ^7^Department of Clinical Research on Social Recognition and Memory, Research Center for Child Mental Development, Kanazawa University, Kanazawa, Japan

**Keywords:** autism spectrum disorders, android robot, motion, sensory sensitivity, sensation seeking

## Abstract

Android robots are employed in various fields. Many individuals with autism spectrum disorders (ASD) have the motivation and aptitude for using such robots. Interactions with these robots are structured to resemble social situations in which certain social behaviors can occur and to simulate daily life. Considering that individuals with ASD have strong likes and dislikes, ensuring not only the optimal appearance but also the optimal motion of robots is important to achieve smooth interaction and to draw out the potential of robotic interventions. We investigated whether individuals with ASD found it easier to talk to an android robot with little motion (i.e., only opening and closing its mouth during speech) or an android robot with much motion (i.e., in addition to opening and closing its mouth during speech, moving its eyes from side to side and up and down, blinking, deeply breathing, and turning or moving its head or body at random). This was a crossover study in which a total of 25 participants with ASD experienced mock interviews conducted by an android robot with much spontaneous facial and bodily motion and an android robot with little motion. We compared demographic data between participants who answered that the android robot with much motion was easier to talk to than android robot with little motion and those who answered the opposite. In addition, we investigated how each type of demographic data was related to participants' feeling of comfort in an interview setting with an android robot. Fourteen participants indicated that the android robot with little motion was easier to talk to than the robot with much motion, whereas 11 participants answered the opposite. There were significant differences between these two groups in the sensory sensitivity score, which reflects the tendency to show a low neurological threshold. In addition, we found correlations between the sensation seeking score, which reflects the tendency to show a high neurological threshold, and self-report ratings of comfort in each condition. These results provide preliminary support for the importance of setting the motion of an android robot considering the sensory traits of ASD.

## Introduction

An android robot is a robot whose appearance and movements resemble those of an actual human. With recent rapid advances in technology, android robots can exhibit facial expressions (e.g., smiling, nodding, and brow movements) during speech and provide subtle non-verbal cues. Such robots are employed in various fields, such as nursing, education, and medical care. Many people have a sense of curiosity and security toward them. Android robots are expected to perform jobs that people originally performed as well as jobs that people cannot perform.

Individuals with autism spectrum disorders (ASD) often achieve a higher degree of task engagement through interactions with humanoid robots than through interactions with humans (1–8). This tendency is also true in their interactions with android robots. There is a growing body of literature indicating that many individuals with ASD have the motivation and aptitude for using android robots (9–12). Using a robot allows researchers to control and replicate a scene that facilitates smooth and accurate conversation despite participants' reactions, which allows a more structured and standardized intervention. Unlike human beings, android robots that operate within predictable and lawful systems provide a highly structured learning environment for individuals with ASD, facilitating their focus on relevant stimuli. Structured interactions with a robot such as interview training can simulate social situations in which certain social behaviors can occur, and daily life (2, 10, 11).

The term “uncanny valley” refers to people's response to a human-like artifact abruptly shifting from high affinity to revulsion when the artifact approaches but fails to attain human appearance (13). The concept of the uncanny valley is the proposed relationship between the humanness of an entity and the perceiver's affinity for it; this suggests that android robots that appear almost, but not exactly, like real human beings elicit uncanny, or strangely familiar, feelings of eeriness and revulsion in observers. However, research indicates that individuals with ASD do not show the uncanny valley effect (14, 15). In contrast, individuals with ASD are suggested to have an affinity for android robots.

To make a robot easy to talk to, the impression of the robot is important. In human-robot interaction, the impression of the robot is affected not only by the optimal appearance but also the optimal motion of the robot (16). The impression of robots is crucial to the success of robot-assisted therapy, such as job interview training for individuals with ASD. Considering that individuals with ASD have strong likes and dislikes (17), ensuring the optimal motion of robots is important to ensure smooth interaction and fully draw out the potential of robotic intervention. Very little is known about how the motion of robots is related to the incentive for individuals with ASD to engage in interventions with robots.

In this study, we investigated the comfort levels of individuals with ASD when talking to a robot with little motion (i.e., only opening and closing its mouth during speech) vs. a robot with spontaneous facial and bodily motions (i.e., in addition to opening and closing its mouth during speech, moving its eyes from side to side and up and down, blinking, deep breathing, and turning or moving its head or body at random). We compared demographic data between participants who answered that the android robot with much motion was easier to talk to than the android robot with little motion and those who answered the opposite. In addition, considering that comfort with an android robot is an important element for the ease of talking to it, we investigated how each type of demographic data was related to this sense of comfort in the interview setting for individuals with ASD. A greater understanding of this relationship could provide insight into developing therapeutic interventions with android robots such as interview training for individuals with ASD.

## Materials and Methods

### Participants

The present study was approved by the ethics committee of Kanazawa University. Participants were recruited by flyers that explained the content of the experiment. In total, 25 individuals with ASD (13–35 years; 3 females, 22 males) took part in the study. After receiving a complete explanation of the study, all participants and their guardians agreed to participate. Written informed consent was obtained from participants and/or from minor participants' legal guardian for the publication of any potentially identifiable images or data included in this article. All participants provided written informed consent. The inclusion criteria included (1) having a diagnosis of ASD based on the Diagnostic and Statistical Manual of Mental Disorders, Fifth Edition (DSM-5) (18), from the supervising study psychiatrist; (2) IQ ≥ 70; and (3) not taking medication.

The exclusion criteria for the ASD group were medical conditions associated with ASD (e.g., fragile X syndrome, Rett syndrome, and Shank3). To exclude other psychiatric diagnoses, the Mini-International Neuropsychiatric Interview (19) was administered. At the time of enrollment, the diagnoses of all participants were confirmed by a psychiatrist with more than 15 years of experience in ASD using the criteria in the DSM-5 (18) and standardized criteria taken from the Diagnostic Interview for Social and Communication Disorders (DISCO) (20). The DISCO has been reported to have good psychometric properties (21).

All participants completed the Autism Spectrum Quotient-Japanese version (AQ-J) (22), which was used in the evaluation of ASD-specific behaviors and symptoms. The AQ-J is a short questionnaire with five subscales (social skills, attention switching, attention to detail, imagination, and communication). In the AQ-J, participants filled out their rating on the questionnaire paper. Previous work with the AQ-J has been replicated across cultures (23) and ages (24, 25). The AQ is sensitive to the broader autism phenotype (26).

Full-scale IQ scores were measured by the Wechsler Intelligence Scale for Children–Fourth Edition, the Wechsler Adult Intelligence Scale–Third Edition or the Japanese Adult Reading Test (JART) (27), which is a standardized cognitive function test to estimate the premorbid IQ of examinees with cognitive impairments. The JART has good validity for measuring IQ, and its results are comparable to those of the Wechsler Adult Intelligence Scale–Third Edition (27). In the Wechsler Intelligence Scale for Children–Fourth Edition, the Wechsler Adult Intelligence Scale–Third Edition, and the Japanese Adult Reading Test (JART), a psychologist interviewed each participant and wrote down the response in the printed form.

The severity of social anxiety symptoms was measured using the Liebowitz Social Anxiety Scale (LSAS) (28). This clinician-administered scale consists of 24 items, including 13 items that describe performance situations and 11 items that describe social interaction situations. Each item was separately rated for “fear” and “avoidance” using a 4-point categorical scale. According to receiver operating curve analyses, an LSAS score of 30 is correlated with minimal symptoms and is the best cutoff value for distinguishing individuals with and without social anxiety disorder (29). In the LSAS, participants filled out their rating on the questionnaire paper.

The Adolescent/Adult Sensory Profile (AASP) is a self-report questionnaire measuring sensory processing in individuals aged 11 years and older (30). In the AASP, participants filled out their rating on the questionnaire paper. The internal consistency coefficients of the AASP range from 0.64 to 0.78 for the quadrant scores. In this study, before the experiment, the participants indicated how often they exhibited certain behaviors related to sensory experiences on a scale ranging from “almost never” (score of 1) to “almost always” (score of 5). The four quadrants of sensory processing examined by the AASP are low registration, sensation seeking, sensory sensitivity, and sensation avoiding. Low registration refers to the tendency to show a high neurological threshold and passive behavioral responses and is associated with sensory bluntness, where even strong sensory stimuli may go unnoticed. This tendency is associated with a delay in response to sensory stimuli. The sensation-seeking score refers to the tendency to show a high neurological threshold and active behavioral responses. Sensation seeking requires specific sensory stimuli to satisfy a high neurological threshold and stabilize an easily bored state without specific sensory stimuli. The sensory sensitivity score refers to the tendency to show a low neurological threshold, receive strong stimuli, and feel pain after exposure to even mild sensory stimuli with passive behavioral responses. The sensation-avoiding score refers to the tendency to show low neurological thresholds and avoid unpleasant sensory stimuli with active behavioral responses. As the AASP does not categorize responses according to individual perceptual domains (such as auditory, visual, or tactile), a perceptual domain analysis was not performed in this study.

### Robotic System

**Figure 1:**
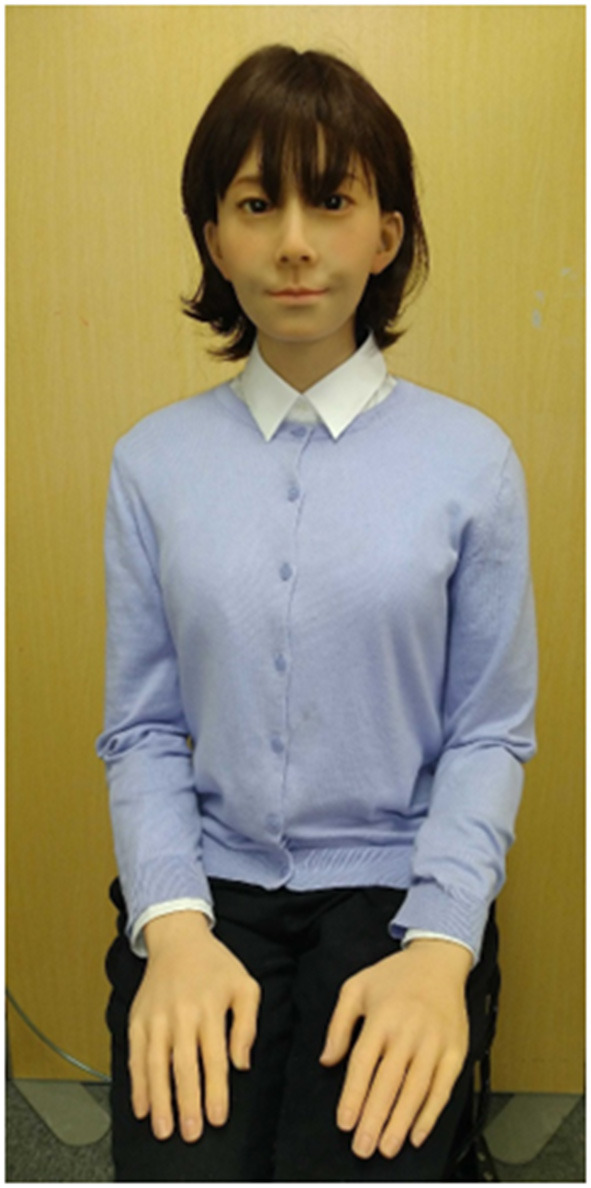
A-Lab Android ST.

The android robot used in this study was A-Lab Android ST (Figure 1) (A-Lab Co., Ltd. Chiyoda-ku, Tokyo, Japan.), which is a female humanoid robot with an appearance similar to that of a real person. Its artificial body has the same proportions, facial features, hair color, and hairstyle as a human. The synthesized voice of the android robot is also similar to that of an actual person. To elicit the belief that the robot behaved and responded autonomously without fail, we adopted a remote-control system similar to that conventionally used in robotics studies (31). The android robot incorporated changes in facial expression (i.e., smiling, nodding, and eyebrow movements) during speech. The first, third, fourth, sixth, and seventh authors and three collaborators checked the combined speech and motions and confirmed that they would not be unnatural or uncanny for participants.

### Procedure

**Figure 2:**
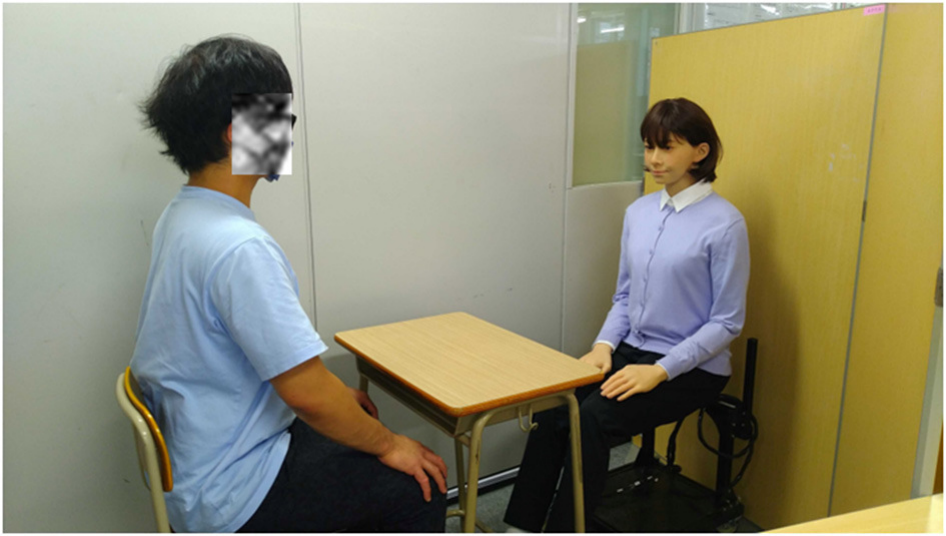
An example of a mock interview by android robot.

This was a crossover study wherein the participants experienced a mock interview conducted by an android robot with much motion or with little motion (Supplementary Video 1). Please see Supplementary Material 1 and Supplementary Video 1 for the setting of the android robot with much motion. In our preliminary experiment, we confirmed that the much-motion setting is an easy conditions for individuals with typical development to talk to a robot. Figure 2 provides an example of a mock interview conducted by an android robot. (The person in Figure 2 provided written informed consent to publish this image.) The android robot was operated by the researcher seated in a different room. During the intervention, when a button was pushed by the researcher, the android robot began to speak following prepared scripts. The researcher monitored the answers given by the participants *via* video. The scripts of the mock interviews varied slightly across sessions to promote engagement but followed the same basic structure. (Examples of scripts are provided in Supplementary Material 2) The questions asked in the interview for the participants were the same as those used in previous studies (9, 11, 32) targeting individuals with ASD. Through these experiences, we realized these questions are relevant. The participant did not know in advance how the setting of the android robot would change.

The trial procedures were conducted for two consecutive time periods. To reduce sequence effects, we counterbalanced the trial conditions between the two groups. The participants in the first group (Group 1; *n* = 13) experienced a mock interview by the android robot with much motion in Period 1 and experienced a mock interview by the android robot with little motion in Period 2, while Group 2 participants (*n* = 12) were in the opposite conditions. The average duration of each mock interview was ~10 min. The break between the two interviews was about 10 min. In this study, the order of questions asked was the same between mock interviews conducted by the android robot with much motion and with little motion. The durations of the interviews were also similar. In both conditions, 22 identical questions were included in the same order, while two questions were followed by phrases to elicit further response. Different questions were chosen to be followed by these phrases depending on the condition.

No participant faced any technical difficulties during the mock interview with the android robot.

After completing both trial conditions, participants answered a question about the robots that they encountered: “In which setting was the android robot easier to talk to?”

In addition, considering that comfort with an android robot is an important element for ease of talking to the robot, as soon as participants completed a mock interview by the android robot with each condition, they completed questionnaires designed to evaluate their comfort in the interview setting in both conditions (much robot motion and little robot motion) (33). The participants evaluated their comfort with the android robot in terms of their feeling at ease, their feeling relieved, their sense of comfort, the robot being friendly, and the robot seeming kind, all on scales of 1 = not at all to 7 = very. Details are presented in Supplementary Material 3. A total score was also calculated by summing the scores for these individual items.

### Data Analysis

We performed statistical analysis using SPSS version 24.0 (IBM, Armonk, NY, USA). Shapiro–Wilk-tests were performed separately for participants who answered that the android robot with much motion was easier to talk to and those who answered that android robot with little motion was easier to talk to. The AQ-J, full-scale IQ score, LSAS, and AASP were found to be normally distributed. Details are presented in Supplementary Material 4. On the other hand, normality was not confirmed for age and some comfort scores (i.e., feeling at ease, sense of comfort, and feeling kind toward the android robot with little motion, and for sense of comfort and feeling kind toward the android robot with much motion). We compared the full-scale IQ score, AQ-J score, LSAS score, and AASP subscore between participants who answered that the android robot with little motion was easier to talk to than the android robot with much motion and those who answered the opposite using a *t*-test. Similarly, based on this criteria, we compared age between participants using Mann-Whitney *U*-test. We used Spearman's rank correlation test to explore the relationships between comfort score (i.e., self-report ratings about comfort in the interview setting) for the android robot with much motion and age, full-scale IQ score, AQ-J score, LSAS score, and AASP subscore. We performed the same analysis for the android robot with little motion.

## Results

In total, 25 individuals with ASD took part in the study. All participants completed the experimental procedure and the questionnaires. We confirmed that all participants noticed a difference between much and little motion conditions after completing both trial conditions. Fourteen participants answered that the android robot with little motion was easier to talk to than the android robot with much motion, whereas 11 participants answered the opposite. There were no differences in age, full-scale IQ score, AQ-J score, LSAS score, low registration score, sensation-seeking score, or sensation-avoiding score between participants who answered that the android robot with little motion was easier to talk to than the android robot with much motion and those who answered the opposite.

Conversely, there were significant differences in the sensory sensitivity score between these two groups. Multiple comparison corrections were performed for the AASP subscales using the Bonferroni method. The significance level after correction using the Bonferroni method was 0.0125. Therefore, the *p*-value for sensory sensitivity was not significant. Regarding the power of sensory sensitivity, we calculated 1-β and found it to be 0.654. In general, this is a relatively low value. However, the effect size is high (Cohen's *d* = 0.973). The effect size is independent of sample size. Significant findings were followed up with the examination of effect size in sensory sensitivity. To examine the possibility that the order of the condition is related to the participants' preference for the amount of movement of the robot, we performed a Fisher's exact probability test. The results were not significant (*p* = 0.69), indicating that the order of execution and robot choice were independent. Details are presented in Table 1.

**Table 1:** Descriptive statistics of participants who preferred the android robot with much motion and the android robot with little motion.

	**Participants who found the android robot with much motion easier to talk to (M, SEM)**	**Participants who found the android robot with little motion easier to talk to (M, SEM)**	**Statistics**	
			***U* or *t* or** **χ^2^**	**df**	***p***
Age	21.00 (5.55)	20.21 (4.49)	*U* = 80.000		0.893
Gender (Male:Female)	9:2	13:1	χ^2^ = 0.711	1	0.399
Full-scale IQ	92.18 (3.66)	95.57 (3.02)	*t* = −0.722	23	0.478
AQ-J	24.27 (2.11)	28.57 (1.64)	*t* = −1.638	23	0.115
LSAS	58.27 (7.20)	55.29 (9.20)	*t* = 0.245	23	0.809
**AASP**					
Low registration	38.45 (2.36)	41.86 (2.88)	*t* = −0.879	23	0.388
Sensation seeking	36.64 (2.11)	43.07 (2.96)	*t* = −1.678	23	0.107
Sensory sensitivity	32.27 (2.53)	42.14 (3.02)	*t* = −2.415	23	0.024^*^
Sensation avoiding	35.55 (1.56)	42.64 (3.06)	*t* = −1.904	23	0.069

*M, mean; SEM, standard error of the mean; SD, standard deviation; AQ-J, autism spectrum quotient, Japanese version. In the AQ-J, higher scores reflect a greater number of ASD-specific behaviors; LSAS, Liebowitz Social Anxiety Scale; AASP, Adolescent/Adult Sensory Profile*.

* *p < 0.05*.

**Figure 3:**
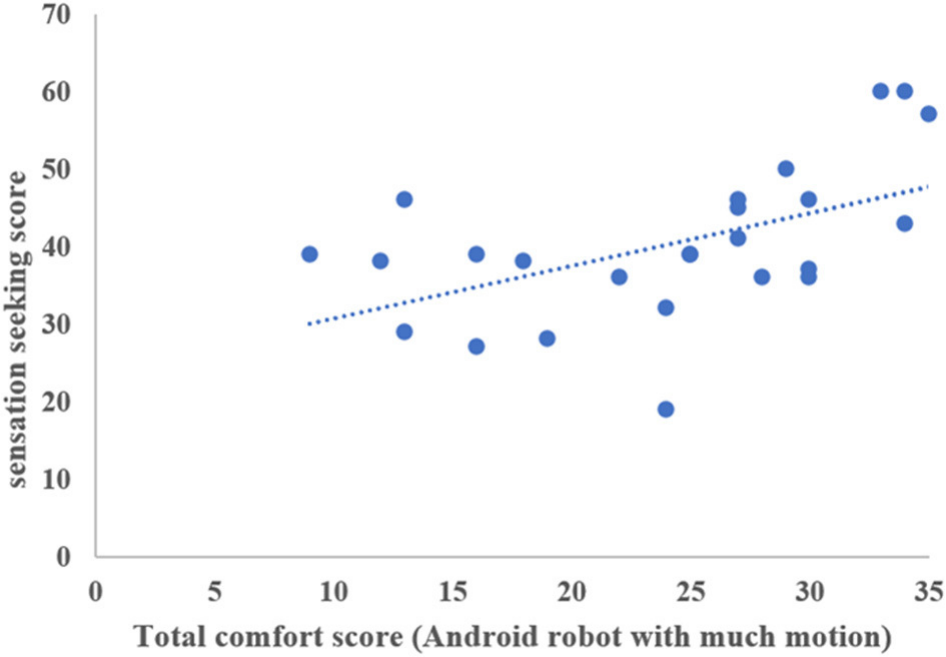
The relationship between total comfort score in android robot with much motion and sensation seeking score.

**Figure 4:**
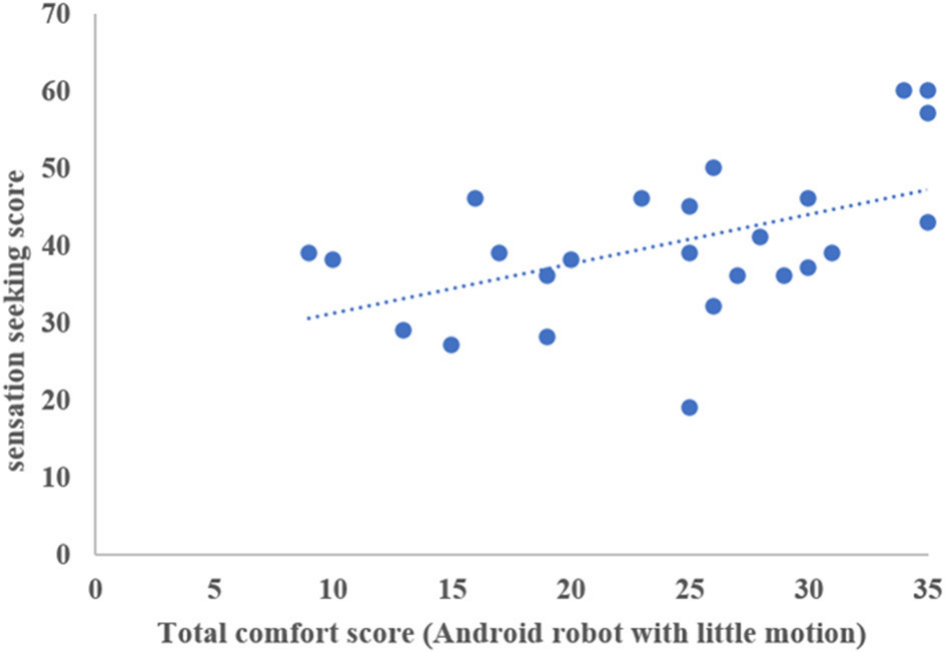
The relationship between total comfort score in android robot with little motion and sensation seeking score.

We found no correlations between the total comfort score for the android robot with much motion and age, full-scale IQ score, AQ-J score, or LSAS score. We also found no correlations between the total comfort score for the android robot with little motion and age, full-scale IQ, AQ-J score, or LSAS score. Conversely, we found correlations between the sensory traits of participants and their comfort scores. Notably, Spearman's rank correlation test revealed significant correlations between the sensation seeking score and self-report ratings of feeling at ease (*r* = 0.527, *p* = 0.007), feeling relieved (*r* = 0.416, *p* = 0.039), perceiving the robot as comforting (*r* = 0.536, *p* = 0.006), perceiving the robot as friendly (*r* = 0.432, *p* = 0.031), perceiving the robot as kind (*r* = 0.401, *p* = 0.047), and total score (*r* = 0.522, *p* = 0.007) for the android robot with much motion. Additionally, Spearman's rank correlation test revealed significant correlations between sensation seeking and self-report ratings of feeling at ease (*r* = 0.411, *p* = 0.041), feeling relieved (*r* = 0.523, *p* = 0.007), perceiving the robot as comforting (*r* = 0.539, *p* = 0.005), perceiving the robot as friendly (*r* = 0.465, *p* = 0.019), perceiving the robot as kind (*r* = 0.433, *p* = 0.031), and total score (*r* = 0.470, *p* = 0.019) for the android robot with little motion. Details about the relationship between the sensory trait and comfort scores in each condition are presented in Table 2 and Figures 3, 4.

**Table 2:** Correlations: comfortableness toward android robot with much motion or little motion and demographic data.

**Item**	**Feeling at ease**	**Feeling relieved**	**Comfort**	**Being friendly**	**Seeming kind**	**Total**
**Android robot with much motion**						
Low registration	0.317	0.212	0.271	0.246	0.208	0.274
Sensation seeking	0.527^**^	0.416^*^	0.536^*^	0.432^*^	0.401^*^	0.522^**^
Sensory sensitivity	0.321	0.385^*^	0.281	0.383	0.222	0.376
Sensation avoiding	0.227	0.417^*^	0.240	0.402^*^	0.194	0.350
**Android robot with little motion**						
Low registration	0.253	0.280	0.426^*^	0.418^*^	0.400^*^	0.399^*^
Sensation seeking	0.411^*^	0.523^**^	0.539^*^	0.465^*^	0.433^*^	0.470^*^
Sensory sensitivity	0.392	0.460^*^	0.487^*^	0.472^*^	0.323	0.456^*^
Sensation avoiding	0.460^*^	0.410^*^	0.495^*^	0.493^*^	0.394	0.522^**^

* *p < 0.05*,

** *p < 0.01*.

## Discussion

In this study, 14 out of 25 high-functioning individuals with ASD answered that the android robot with little motion was easier to talk to than the android robot with much motion. On the other hand, 11 participants answered the opposite. Our data suggest that not all individuals with ASD felt that the robot with simple movements was easier to talk to than the robot with complex movements. Importantly, individuals with higher levels of reported sensory sensitivity found it easier to talk to the android robot with little motion. In addition, individuals with ASD who showed specific sensory traits, especially higher sensation seeking scores, felt comfort with the android robot in both conditions (i.e., much motion and little motion). While our sample sizes were somewhat small for statistical comparisons, our quantitative data indicated the abovementioned trends.

Previous research suggests that individuals with ASD gravitate toward simple behaviors (4, 34–37). Robins et al. (35) concluded that robots designed to interact with individuals with ASD should be less detailed and less visually complex than humans while still conforming to the humanoid form. Given these studies, we expected that most individuals with ASD would find it easier to talk to an android robot with little motion than one with much motion. However, we found that this was not the case for all individuals with ASD. Additionally, given these studies, we also expected that many individuals with ASD, especially those with more severe autistic traits, would prefer plain, visibly mechanical robots over those with a more human-like appearance. However, we found no correlations between comfort scores for the android robot in each condition and AQ. However, we found correlations between comfort scores and sensory trait scores, especially the sensation seeking score. Considering a user's sensory trait—especially the sensation seeking trait, which refers to individual differences in motivation for intense and unusual sensory experiences—may be important in setting the optimal motion of robots for interventions with individuals with ASD. When setting spontaneous facial and bodily motion, simple motions may be not always better for individuals with ASD.

In their guidelines for humanoid robot designs, Ricks and Colton (4) state that individuals with ASD could begin therapy with a simplistic robot, and as they become more comfortable, it may be useful to introduce a more realistic robot. The results of our study suggest that considering the setting of motion is also important for effective robotic intervention. In most previous studies using robots for individuals with ASD, the setting of motion was consistent during the interventions. To ensure the success of robotic intervention, setting motion considering the sensory traits of participants exploit the potential abilities of an android robotic intervention to the fullest extent. Thus, sophisticated guidelines considering not only optimal appearance but also motion setting may be necessary for the design of therapeutic robots. Our current results may contribute to this process.

In this preliminary study, participants had only one interaction with the android robot in each setting. We expect that not only the optimal appearance but also the optimal motion change as participants become acclimated to interacting with the robot. While the current study was not able to test habituation effects, it represents one of the first systematic investigations of the optimal setting of motion to make it easier for individuals with ASD to talk to android robots. In future work, it would be important to evaluate habituation effects with android robots by observing interactions over an extended period of time. In addition, considering that individuals with ASD have restricted interests, it is important to know which parts of the robot's body they prefer to see move.

We acknowledge several limitations of our study. First, our sample size was relatively small. Larger sample sizes are necessary to provide more meaningful data. In addition, most of our participants were male. Future research should include more female participants. Second, our interview was relatively short; however, we judged that 10 min per session would be appropriate to meet the specific needs of individuals with ASD. In addition, all our participants were able to complete the trial. Third, we included only individuals with ASD. Thus, it is unclear whether these findings are specific to individuals with ASD. In addition, to further clarify the relationship between optimal motion in android robots and sensory traits, it is important to study individuals without ASD and compare their data with those of individuals with ASD. All participants received scores for the Wechsler Intelligence Scale for Children–Fourth Edition, the Wechsler Adult Intelligence Scale–Third Edition, or the Japanese Adult Reading Test (JART) a half year before the experiment. At the time of this experiment, the Japanese government had declared a state of emergency due to the spread of COVID-19, and therefore we could not recruit control participants and ask participants to undergo additional face-to-face IQ tests for the experiment. Finally, the within-subject design might have been subject to the “carryover effect”, which may have affected the results.

This study revealed that the setting of android robots' spontaneous facial and bodily motion influences the ease of talking to the robots for individuals with ASD and that individuals with higher levels of reported sensory sensitivity found it easier to talk to the android robot with little motion. Individuals with ASD have strong likes and dislikes (17), and if a patient dislikes a therapeutic robot, it may not be possible to perform therapy. Despite this study's limitations, it suggests that setting not only the optimal appearance but also the optimal motion of an android robot considering the sensory traits of ASD is important to exploit the full potential of robotic interventions.

## Data Availability Statement

The raw data supporting the conclusions of this article will be made available by the authors, without undue reservation.

## Ethics Statement

The studies involving human participants were reviewed and approved by the Ethics Committee of Kanazawa University. Written informed consent to participate in this study was provided by the participants' legal guardian/next of kin.

## Author Contributions

HK designed the study, conducted the experiment, carried out the statistical analyses, analyzed and interpreted the data, and drafted the manuscript. TM, YY, YM, KT, HI, MM, and MK conceived of the study, participated in its design, assisted with data collection and the scoring of behavioral measures, analyzed and interpreted the data, were involved in drafting the manuscript, and critically revising it for important intellectual content. MM was involved in approving the final version to be published. All authors read and approved the final manuscript.

## Funding

This work was supported in part by Grants-in-Aid for Scientific Research from the Japan Society for the Promotion of Science (21H04418 and 20H05575) and JST, Moonshot R&D Grant Number JPMJMS2011.

## Conflict of Interest

The authors declare that the research was conducted in the absence of any commercial or financial relationships that could be construed as a potential conflict of interest.

## Publisher's Note

All claims expressed in this article are solely those of the authors and do not necessarily represent those of their affiliated organizations, or those of the publisher, the editors and the reviewers. Any product that may be evaluated in this article, or claim that may be made by its manufacturer, is not guaranteed or endorsed by the publisher.
