# Supplementary material for: Differences in the Optimal Motion of Android Robots for the Ease of Communications Among Individuals With Autism Spectrum Disorders
Source: Front Psychiatry. 2022 Jun 3;13:883371. doi: 10.3389/fpsyt.2022.883371 (PMC9203835; doi:10.3389/fpsyt.2022.883371)
Supplement: Supplementary file 2 [file Data_Sheet_1.DOCX]

Supplementary Material

# Supplementary Material 1

The android robot with much motion performed eye movement and blinking at the following frequencies in periods of one to four seconds at random:

Performing eye movement from side to side (10%)

Performing eye movement up and down (10%)

Blinking one time quickly (10%)

Blinking two times in a row (20%)

Blinking one time slowly (50%)

In addition, the robot performed deep breathing and turned or moved its head or body at random in the following frequencies in periods of one to four seconds at random:

Deep breathing (40%)

Turning its head and body to the left (10%)

Turning its head and body to the right (10%)

Moving its head and body forward (10%)

Moving its head and body backward (10%)

Turning its head diagonally to the left and its body to the left (10%)

Turning its head diagonally to the left and its body to the right (10%)
