# Supplementary material for: Differences in the Optimal Motion of Android Robots for the Ease of Communications Among Individuals With Autism Spectrum Disorders
Source: Front Psychiatry. 2022 Jun 3;13:883371. doi: 10.3389/fpsyt.2022.883371 (PMC9203835; doi:10.3389/fpsyt.2022.883371)
Supplement: Supplementary file 4 [file Data_Sheet_3.DOCX]

Supplementary Material 3 (S3)

Questionnaires designed to evaluate their comfort in the interview setting

1． I feel at ease with this robot.

2． I feel relieved with this robot.

3． The robot looks as if it could comfort me.

4． The robot moves in a friendly manner.

5． The robot looks kind.
