# Supplementary material for: Differences in the Optimal Motion of Android Robots for the Ease of Communications Among Individuals With Autism Spectrum Disorders
Source: Front Psychiatry. 2022 Jun 3;13:883371. doi: 10.3389/fpsyt.2022.883371 (PMC9203835; doi:10.3389/fpsyt.2022.883371)
Supplement: Supplementary file 5 [file Data_Sheet_4.DOCX]

**Supplementary Material 4 (S4)**

Shapiro-Wilk test for normality of participants who answered that the android robot with much motion was easier to talk to and those who answered that android robot with little motion was easier to talk to.

|  | Android robot with much motion | Android robot with little motion |
| --- | --- | --- |
| Age | .84* | .94 |
| Full-scale IQ | .97 | .98 |
| AQ-J | .93 | .97 |
| LSAS | .94 | .91 |
| AASP |  |  |
| Low Registration | .97 | .98 |
| Sensation Seeking | .93 | .91 |
| Sensory Sensitivity | .92 | .93 |
| Sensation Avoidance | .96 | .93 |

**p* <0.05

*Note.* AQ-J: autism spectrum quotient, Japanese version. In the AQ-J, higher scores reflect a greater number of ASD-specific behaviors.

LSAS = Liebowitz Social Anxiety Scale.

AASP= Adolescent/Adult Sensory Profile
